# Awareness and Perception Regarding Eye Donation in Students of a Nursing College in Bangalore

**DOI:** 10.4103/0970-0218.51235

**Published:** 2009-04

**Authors:** Anita Gupta, Sudhir Jain, Tanu Jain, Karan Gupta

**Affiliations:** Center of Epidemiology, National Institute of Communicable Diseases, Delhi, India

**Keywords:** Corneal transplantation, diminished vision, eye donation, nursing students

## Abstract

**Context:**

Corneal diseases constitute a significant cause of visual impairment and blindness in the developing world. The number of corneal transplants done is far less than the actual requirement in India. This is largely due to the inadequate number of corneas collected. Well-informed nursing students could be expected to influence eye donation rates.

**Aims:**

To assess the awareness and perception of 188 first- and second-year nursing students towards eye donation in Bangalore.

**Settings and Design:**

Cross-sectional study design.

**Materials and Methods:**

A pretested, semi-structured questionnaire.

**Statistical Analysis Used:**

Data were analyzed using the Epi-Info software package, Version 6.04.

**Results:**

The majority (96.8%) of students knew that eyes can be donated after death but only 38.2% knew that the ideal time of donation was within 6 hours of death. Most participants (85.1%) were either willing or had already donated their eyes. Nobility in the act of eye donation was the main motivational force for eye donation according to 85.6% of students. Perceived reasons for not pledging eyes by the students were: the unacceptable idea of separating the eyes from the body (67.9%), lack of awareness (42.8%), objection by family members (28.5%), and unsuitability to donate because of health problem (10.7%).

**Conclusion:**

This study revealed that nursing students were well aware of eye donations and most of them were inclined to sign-up for eye donation. The perceived reasons for not donating eyes need to be considered while creating awareness about eye donation in the community. The nursing students could be actively involved as volunteers in eye donation campaigns and they can act as counsellors for eye donors. They can also contribute by participating in creating awareness and motivating people to become eye donors.

## Introduction

Corneal diseases are a significant cause of visual impairment and blindness in the developing world. The major causes of corneal blindness include trachoma, corneal ulceration following xerophthalmia due to vitamin A deficiency, ophthalmia neonatorum, and the use of harmful traditional medicines, onchocerciasis, leprosy, and ocular trauma.(1–3)

The Andhra Pradesh Eye disease study (APEDS)(4) reported the prevalence of corneal blindness at 0.13% (95% CI: 0.06-0.24), constituting 9% of all blindness. APEDS also suggested a significant burden of corneal blindness in the rural population of Andhra Pradesh, of which 95% was avoidable. Although strategies to prevent corneal blindness are likely to be more cost-effective, visual rehabilitation by corneal transplantation remains a major treatment option for restoring sight in those who already have corneal blindness.(4)

Approximately 18.7 million people are blind in India(5) and 190,000 are blind from bilateral corneal disease. Every year, another 20,000 join the list.(6) The late Dr. Muthiah started the very first eye bank in India and he successfully performed the first corneal transplant in 1948.(7) Even after more than 50 years, patients waiting for corneal transplants constitute a considerable backlog, which continues to grow. The need, therefore, is to educate the masses about eye donation in an effort to increase the procurement of corneas.

According to the Eye Bank Association of India, the current cornea procurement rate in India is 22,000 per year. It is estimated that a significant proportion of donor corneas are unsuitable for corneal transplantation.(8) Based upon our current ratio of available safe donor eyes, we would need 277,000 donor eyes to perform 100,000 corneal transplants in a year in India.(9) There is approximately a 20-fold increase from the donor eyes available now. A shortage of transplantable corneas is common and has been the subject of much attention. To increase procurement of corneas, raising the level of public education on eye donation is an important first step. Soliciting for actual eye donation at the time of death is a necessary and accepted practice.(9) Though the factors affecting procurement of corneas and the public attitude towards eye donation have recently received attention in the developed world,(10) not much has been published from the developing world.

Nursing students are the future health care providers for the community. They are from different educational backgrounds with a scientific base and have an inherent inclination to serve mankind. With this background, they were admitted into this study with the objective of assessing their knowledge and attitude on eye donation.

Well-informed nursing students could be expected to influence eye donation rates. The education of para-medical early in their courses may lead to better procurement rates for donor organs. This study was designed to assess the perception of nursing students towards eye donation and their willingness to pledge eyes.

## Materials and Methods

**Type of study:** Cross-sectional study design

**Period of study:** May 2008

**Study site:** Florence College of nursing, Kalyan Nagar, Bangalore, Karnataka

**Sample size:** 188 first- and second-year nursing students

A pretested, semi-structured questionnaire was self-administered for collecting the necessary information after obtaining informed consent. The questionnaire contained questions on demographic details, awareness regarding eye donation, reasons for donating and not donating eyes, intention to donate eyes, and sources of information.

The data were entered and analyzed using the Epi-info software package, Version 6.04. Data was expressed in proportion.

**Ethics:** The study was carried according to the ethical guidelines for biomedical research on human subjects (2000).

## Results

Out of 188 students, 106 (56.4%) were males and 82 (43.6%) were females. Age varied from 18 to 21 years old with 41 (21.8%) students who were 18 years old, 100 (53.3%) students who were 19 years old, 39 (20.7%) students who were 20 years old, and 8 (4.2%) students who were 21 years old.

Of the 188 students, it was observed that 102 (96.2%) males and 80 (97.5%) females knew that eyes can be donated after death; that they should ideally be donated within 6 hours of death was known to 72 (38.2%) of the 182 students. The contact place for donation was known to only 62 (32.9%) of 188 students. The majority of the participants, 108 (85.1%) of 188 students, were either willing to donate eyes or had already pledged to donate their eyes. No major difference according to gender was observed in the responses [Table 1].

**Table 1:** Responses to the questionnaire on eye donation (n=188)

Responses	Male	% (n=106)	Females	% (n=82)
Eyes can be donated after death	102	96.2	80	97.5
Donated eyes can be used for corneal grafting	80	75.4	60	73.1
Ideal time for donating eyes is within 6 hours after death	40	37.7	32	39
Knows a person who has donated eyes	2	1.9	1	1.2
Knows someone who has received a donated eye	0	0	0	0
Knows contact place for eye donation	37	34.9	25	30.5
Knows there is an eye shortage in India	91	85.8	68	82.9
Willing to donate eyes	62	58.4	46	56
Already pledged to donate	33	31.2	19	23.2
Awareness about selling and buying of donor eyes	0	0	0	0
Agree to sell donor eyes	0	0	0	0

Television was the most common source of information on eye donation for 145 (77.1%) students, followed by the newspaper for 136 (72.8%) students and magazines for 94 (50%) students [Table 2].

**Table 2:** Source of information on eye donation (n=188)*

Source	Number	Percentage
Television	145	77.1
Newspaper	136	72.8
Magazine	94	50
Poster	47	25
Doctor	52	27.6
Radio	70	38.2
Friends	69	36.7
Family members	28	14.8
Pamphlets	42	22.3
Others	52	27.6

* Multiple responses

Nobility in the act of eye donation was the main motivational force according to 137 (85.6%) of the 160 students. Other major reasons were pleasure to help the blind (77.5%), donated eyes can give vision to a person (71.8%), and influenced after reading an article (71.2%) [Table 3].

**Table 3:** Distribution of perceived reasons for donating eyes by donors (n=160)*

Reason	Number	Percentage**
Eye donation is a noble work	137	85.6
Pleasure to help the blind	124	77.5
Donated eyes can give vision to a person	115	71.8
Influenced after reading an article	114	71.2
A friend or relative has donated an eye	3	1.8
A friend or relative has received a donated eye	0	0
Influenced by any lecture	8	5

* included students who have either pledged or are willing to donate eyes.

** Multiple responses.

Lack of awareness was cited as an important reason for people not donating their eyes among 12 (42.8%) of 28 students. Nineteen (67.9%) of 28 students disliked the idea of separating their eyes from their body and 8 (28.5%) sited objection by family members for not donating their eyes [Table 4].

**Table 4:** Distribution of perceived reasons for not donating eyes (n=28)*

Reason	Number	Percentage
Lack of awareness	12	42.8
Objection by family members	8	28.5
Feels body ill-treated by eye donation	7	25
Dislike of separating eyes from body	19	67.9
Unsuitability to donate eye because of age	12	42.8
Unsuitability to donate eye because of health problems	3	10.7
Religious restrictions in separating eyes from the body after death	3	10.7
Signing eye donation card is like signing death certificate	11	39.2

* Multiple responses

Nearly half of the respondents, 89 out of 188 (47.3%) opined that donor's consent should be mandatory and it should be expressed before death, whereas according to 27 out of 188 (14.4%) students, consent should be mandatory but may be given by another adult family member. According to 56 out of 188 (29.8%) students, consent is not necessary but the eyes can be donated if the donor alone wishes and among 16 out of 188 (8.5%) students, consent is not necessary but eyes can be donated if the family members of the donors wish to do so.

## Discussion

In this study, 96.8% of the students were aware that eyes could be donated after death. In a study among the south Indian population, 50.7% of participants were aware of eye donation.(11) In another study among hospital staff, 97% of them had good to excellent knowledge about transplantation of various human organs.(12,13) Information by mass media could be related to the high level of awareness in our study participants.

A large number of students, 140 (74.4%) out of 188 knew that the donated eye is used for corneal grafting and 32.8% knew that the ideal time for donation is within 6 hours of death. A study on medical and nonmedical students also observed that 79.6% of medical students knew that eyes can be donated after death and 63.3% knew that it should be done within 6 hours.(14) Another study in the general population showed the awareness level on eye donation to be 73.8%.(1) In this study, only 62 (32.9%) out of 188 students knew about the appropriate place for an eye donation.

Our study showed that 159 (84.5%) of 188 participants agreed that there is a shortage of eye donors and 160 (85.1%) of 188 were either willing or had already pledged to donate their eyes. In a study among medical students, 87.8% of the respondents were willing to be eye donors.(14) Another study in the urban population observed that 73.8% were aware of eye donations and only 44.9% were willing to pledge their eyes.(15) Willingness to donate eyes was less (41.5%) even among relatives of post-mortem cases who were aware of eye donation.(16)

The prime reasons cited in the study for eye donation was nobility in the work by 137 (85.6%) and pleasure to help the blind by 124 (77.5%) of the 160 participants. But lack of awareness was the reason for people not donating eyes according to 12 (42.8%) of the 28 respondents. Other reasons for not donating eyes included objection by family members, dislike of disfiguring the body, delaying of religious rites, and religious restrictions. Similar reasons were also reported in other studies.(15,17)

Mandatory consent for donation expressed before the death of the donor should ideally form the basis for eye donation. However, in the case of unavailability of such consent, consent from adult family members of the deceased donor should be obtained for eye donation. In a study done on the responses of relatives of post-mortem cases, it was revealed that out of the potential post-mortem donors, only 44.3% of relatives of such cases gave consent for donation after intensive counseling.(17) Mass media in the form of television, newspapers, magazines, and posters were important sources of information on eye donation. Other studies also found publicity campaigns and the media to be the major sources of information on this issue.(1,16)

The present study revealed that nursing students were well aware of eye donation and most of them were inclined to pledge for eye donation. The perceived reasons for not donating eyes need to be considered while creating awareness about eye donation in the community. The nursing students could be actively involved as volunteers in eye donation campaigns, wherein after proper training in counselling techniques, they can act as counsellors for eye donors. They can also contribute by participating in creating awareness and motivating the people for eye donation during their postings in community medicine.
